# Prognostic Significance of CD4+ and CD8+ Tumor-Infiltrating Lymphocytes in Head and Neck Squamous Cell Carcinoma: A Meta-Analysis

**DOI:** 10.3390/cancers13040781

**Published:** 2021-02-13

**Authors:** Daniele Borsetto, Michele Tomasoni, Karl Payne, Jerry Polesel, Alberto Deganello, Paolo Bossi, James R. Tysome, Liam Masterson, Giancarlo Tirelli, Margherita Tofanelli, Paolo Boscolo-Rizzo

**Affiliations:** 1 Department of ENT, Addenbrookes’ Hospital, Cambridge University Hospital Trust, Cambridge CB2 0QQ, UK; daniele.borsetto@addenbrookes.nhs.uk (D.B.); james.tysome@addenbrookes.nhs.uk (J.R.T.); liam.masterson@addenbrookes.nhs.uk (L.M.); 2 Department of Neuroscience, University of Cambridge, Cambridge CB2 1TN, UK; 3 Otorhinolaryngology-Head and Neck Surgery, Department of Medical and Surgical Specialties, Radiological Sciences and Public Health, University of Brescia, 25123 Brescia, Italy; m.tomasoni022@unibs.it (M.T.); alberto.deganello@unibs.it (A.D.); 4 Institute of Head and Neck Studies and Education, University of Birmingham, Birmingham B15 2TT, UK; 5 Unit of Cancer Epidemiology, Centro di Riferimento Oncologico di Aviano (CRO) IRCCS, 33081 Aviano, Italy; polesel@cro.it; 6 Medical Oncology Unit, Department of Medical Oncology, ASST Spedali Civili di Brescia, 25123 Brescia, Italy; paolo.bossi@unibs.it; 7 Department of Medical, Surgical and Health Sciences, Section of Otolaryngology, University of Trieste, 34149 Trieste, Italy; tirellig@units.it (G.T.); mtofanelli@units.it (M.T.); paolo.boscolorizzo@units.it (P.B.-R.)

**Keywords:** head and neck cancer, tumor-infiltrating lymphocyte, prognosis, TIL, cancer

## Abstract

**Simple Summary:**

Tumor infiltrating lymphocytes (TILs) have been demonstrated as prognostic biomarkers in multiple cancer types. Among the various TIL phenotypic sub-populations, T-cells are most abundant. Several studies have investigated the prognostic value of CD4+ and CD8+ T-cell TILs in head and neck squamous cell carcinoma (HNSCC). In this study we performed a systematic review and meta-analysis of available evidence for CD4+ and CD8+ TIL biomarkers in HNSCC. The primary aim was to investigate the correlation of TIL sub-population levels and overall survival in HNSCC anatomical sub-sites. We demonstrate for the first time that tumor location has a significant impact upon the prognostic utility of CD4+ and CD8+ TILs in HNSCC. Such data is of critical importance when incorporating TIL biomarkers into current prognostic models and clinical practice.

**Abstract:**

Objective: It has been suggested that the presence of tumor-infiltrating lymphocytes (TILs) in the tumor microenvironment is associated with a better prognosis in different types of cancer. In this systematic review and meta-analysis, we investigated the prognostic role of CD4+ and CD8+ TILs in head and neck squamous cell carcinoma (HNSCC). Methods: PubMed, Cochrane, Embase, Scopus, and Web of Science were searched up to September 2020. This study was conducted following the preferred reporting items for systematic reviews and meta-analyses (PRISMA) checklist. Risk ratios from individual studies were displayed in forest plots and the pooled hazard ratios (HR) of death and corresponding confidence intervals (CI) were calculated according to random-effects models. Risk of bias of the included studies was assessed through the Newcastle–Ottawa scale. Results: 28 studies met the inclusion criteria. Studies conducted on HNSCC subsites combined reported a significant reduction in the risk of death for both high CD4+ (HR: 0.77; 95% CI: 0.65–0.93) and high CD8+ TILs (HR: 0.64; 95% CI: 0.47–0.88). High CD4+ TILs were associated with significantly better overall survival among oropharyngeal HNSCC (HR: 0.52; 95% CI: 0.31–0.89), as well as high CD8+ TILS in Human papillomavirus −ve and +ve cancers (HR: 0.39; 95% CI: 0.16–0.93 and HR: 0.40; 95% CI 0.21–0.76 respectively). CD8+ TILs were also associated with improved survival in hypopharyngeal cancers (HR = 0.43 CI: 0.30–0.63). No significant association emerged for patients with cancer of the oral cavity or larynx. Conclusions: The findings from this meta-analysis demonstrate the prognostic significance of CD8+ and CD4+ TILs in HNSCC and variation in tumor subsite warrants further focused investigation. We highlight how TILs may serve as predictive biomarkers to risk stratify patients into treatment groups, with applications in immune-checkpoint inhibitors notable areas for further research.

## 1. Introduction

The survival rate for head and neck squamous cell carcinoma (HNSCC) is poor and depends on the subsite: in Europe, 5-year survival rates were 45% for oral cavity, 39% for the oropharynx, 25% for the hypopharynx, 59% for the larynx [1]. Considering the poor survival rates, improved risk stratification is needed to identify patients at higher risk of recurrence and to tailor treatment in these patients. Cancer biomarkers, either tissue or liquid biopsy-based, are promising tools for detection, assessment of prognosis, and prediction of response to therapy.

HNSCC is associated with pronounced immunosuppressive tumor activity. In addition, immunodeficiency has also been shown to correlate with a poor prognosis [2]. Immunosuppression creates a favorable environment for HNSCC cells to avoid tumor eradication through immunosurveillance [3,4]. The tumor immune microenvironment (TIM) is the immune infiltrate that emerges during tumor growth and several reports have shown it has a major role in cancer progression [5]. The TIM includes various tumor-infiltrating lymphocytes (TILs), which influence cancer invasion and metastasis. TILs have been indicated as a promising prognostic marker in multiple cancer types, demonstrating comparable or even greater prognostic value than conventional TNM staging [6]. Moreover, TILs are considered to be a potential predictive biomarker of targeted and immunotherapy treatment. Current guidelines recommend the scoring of TILs on histopathological specimens (both intra-tumoral and stromal TILs) on a percentage scale [7] and in turn tumors are graded as with low, moderate, or high TILs. However, difficulty arises when defining clinically relevant cut-off values for ‘high’ TILs tumors, particularly in HNSCC where tumor subsite and Human papillomavirus (HPV) status has been shown to have a significant impact [8]. For example, across all HNSCC a TIL score of 70% has been shown as prognostically significant [9]. However, studies of tumor subsites such as the oral cavity or oropharynx report significance with TIL scores as low as 20% [8,10].

It has been recently reported that the phenotypic composition of the lymphocytic infiltrate may hold additional value and insight when using TILs as cancer biomarkers. In solid tumors, T lymphocytes are observed to be the principal component of the TIL compartment [11], of which CD4+ T helper cells and CD8+ cytotoxic T cells play pivotal roles. Research demonstrates that CD4+ T cells, CD8+ T cells, natural killer (NK) cells, M1 phenotype macrophages and dendritic cells (DCs) all have anti-tumoral effects. A coordinated and balanced interaction of these subsets is required to guard the host against a developing tumor [12]. In particular, CD8+ infiltration has been reported to be associated with a favorable prognosis in several malignancies [13]. Recent studies have characterized immune infiltrates in the tumor microenvironment of HNSCC and broadly agree that high levels of tumor immune cell infiltrates correlate with an improved prognosis. However, HNSCC demonstrates significant TIL heterogeneity, further compounded by subsite variation [14]. Direct correlations to survival outcomes have varied with reports disagreeing as to the clinical importance of CD4+ compared to CD8+ T cells [15,16].

Considering the need for robust biomarkers in HNSCC and the contrasting evidence concerning TILs in this cancer group, the aim of this paper was to systematically review and perform a meta-analysis of recent evidence concerning the prognostic significance of CD4+ and CD8+ TIL populations in HNSCC. Unlike previous reports, our aim was to provide particular focus on how tumor subsite and HPV status impacts the utility of a CD4+ CD8+ TIL biomarker in HNSCC.

## 2. Methods

### 2.1. Outcome Measures

The primary outcome of this meta-analysis is defined as the prognostic role of CD4+ and CD8+ cell TIL populations in overall survival (OS, defined as the time from diagnosis or initiation of treatment to patient death, irrespective of cause) in HNSCC patients. A secondary outcome is the impact of tumor subsite (oral cavity, oropharynx, hypopharynx, larynx) and HPV status (as detected by p16 immunohistochemistry, HPV-DNA in situ hybridization or PCR) on the above clinical outcome variable. After an initial scoping review of potential studies, we decided we were unable to include additional disease-specific survival outcome measures, such as disease-free or progression-free survival, due to significant heterogeneity and underreporting of these outcomes in studies.

### 2.2. Search Strategy

This systematic review and meta-analysis were conducted following the preferred reporting items for systematic reviews and meta-analyses (PRISMA) checklist [17]. Medline (via Ovid), Cochrane, Embase (via Ovid), Web of Science (Core Collection) and Scopus were searched from inception through to September 2020. The following keyword search was conducted:

“Head and Neck Neoplasms” OR “Facial Neoplasm” OR “Mouth Neoplasm” OR “Otorhinolaryngologic Neoplasm” OR “Tracheal Neoplasm” OR “head and neck neoplasm” OR “Carcinoma” OR “Squamous Cell” OR “oral cavity neoplasm” OR “Oropharyngeal Neoplasms” OR “Hypopharyngeal Neoplasms” OR “Laryngeal Neoplasms” OR ”squamous cell carcinoma” AND “lymphocytes” OR “tumor-infiltrating” OR “tumor-infiltrating lymphocytes” OR “lymphocyte, tumor-infiltrating” OR “tumor-infiltrating lymphocytes” OR “tumor-infiltrating lymphocyte” OR “tumor-derived activated cells” OR “activated cell, tumor-derived” OR “activated cells, tumor-derived” OR “tumor-derived activated cells” OR “tumor-derived activated cell” AND “prognosis” OR “risk” OR “recurrence” OR “mortality” OR “survival” OR “predict” OR “outcome” OR “significant” OR “impact” OR “detect” OR “relevant”.

**Figure 1:**
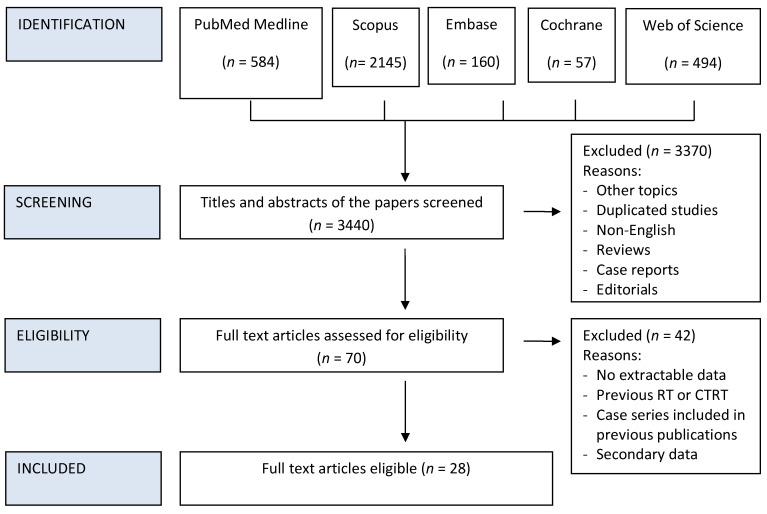
PRISMA Flow chart of study inclusion process.

The reference lists of articles included in this review as well as narrative reviews published in the last 10 years were also manually searched to minimize the risk of missing data. Two authors (DB and MiT) independently screened all titles and abstracts generated by the search and then evaluated the full texts of all the relevant articles identified against the inclusion criteria (Figure 1); a third author (MaT) settled discordances when present. Any disagreement between the assessors on the suitability of articles for inclusion tackled by thorough discussion between assessors, or failing this, by referral to the senior author (PBR).

### 2.3. Selection Criteria

Studies were included in the analysis if they met the following criteria: (1) the study reports the prognostic role of TILs in squamous cell carcinoma of the head and neck region or, specifically, in the oral cavity/oropharynx/larynx/hypopharynx treated with surgery and/or chemoradiation therapy with curative intent; (2) the study uses immunohistochemistry (IHC) to categorize specific subsets of TILs in tumor surgical specimens; (3) the study reports the association of TIL infiltration and patient survival with sufficient survival data to extract hazard ratios (HRs) and 95% confidence intervals CI; (4) the analyzed tissue had not been previously exposed to radiotherapy and/or chemotherapy (5) the study reports specific data for CD8+ TILs in HPV+ and HPV− oropharyngeal SCC. Non-English studies were excluded. Studies containing aggregated data or duplicated data from previously published work were excluded, as were review articles, case reports, editorials, and letters.

### 2.4. Data Extraction and Statistical Analysis

The standard error of the log HR was derived from the log CIs. The pooled HR and corresponding 95% confidence interval (CI) were calculated according to random-effects models of DerSimonian and Laird [18], which incorporates both within-and between-study variability, as a weighted average of the estimated HRs, by giving each study a weight proportional to its precision. Statistical heterogeneity among studies was evaluated using the I² and τ^2^ statistics [18]. Influence analysis was performed when pooled HR were estimated from five or more studies: pooled HR was calculated by omitting one study at a time. Publication bias was assessed through a funnel plot [19]. Two authors (DB, MiT) independently assessed the quality of the included studies with the Newcastle–Ottawa Scale [20].

The results of the meta-analysis were presented graphically using forest plots, plotting the individual paper and pooled HR and 95% CI. 95% CIs were derived from estimated study variances. Statistical significance was defined as *p* < 0.05 (two sided).

## 3. Results

Details on the literature search process are shown in the flow chart of Figure 1. Twenty-eight studies met the eligibility criteria out of the 3440 initially screened citations (Table 1) [15,16,21,22,23,24,25,26,27,28,29,30,31,32,33,34,35,36,37,38,39,40,41,42,43,44,45,46]. Upon contacting the corresponding authors of the papers by Nguyen et al. and Spector et al. it was discovered that there was overlap in patient cohort datasets in certain TIL sub-group analyses presented in these studies. Therefore, to allow the inclusion of valid data we excluded the replicated data from the later published paper of Spector et al., and only included overall HNSCC data from the larger cohort presented by Nguyen et al.

The quality of included studies was high (Newcastle-Ottawa Scale score ≥7) in 21 (75%) of 28 studies, with a median of 8 (interquartile range 7–8). A detailed report on the quality of included studies according to the Newcastle-Ottawa Scale is reported in Supplementary results Table S1.

### 3.1. CD4+ and Overall Survival

**Figure 2:**
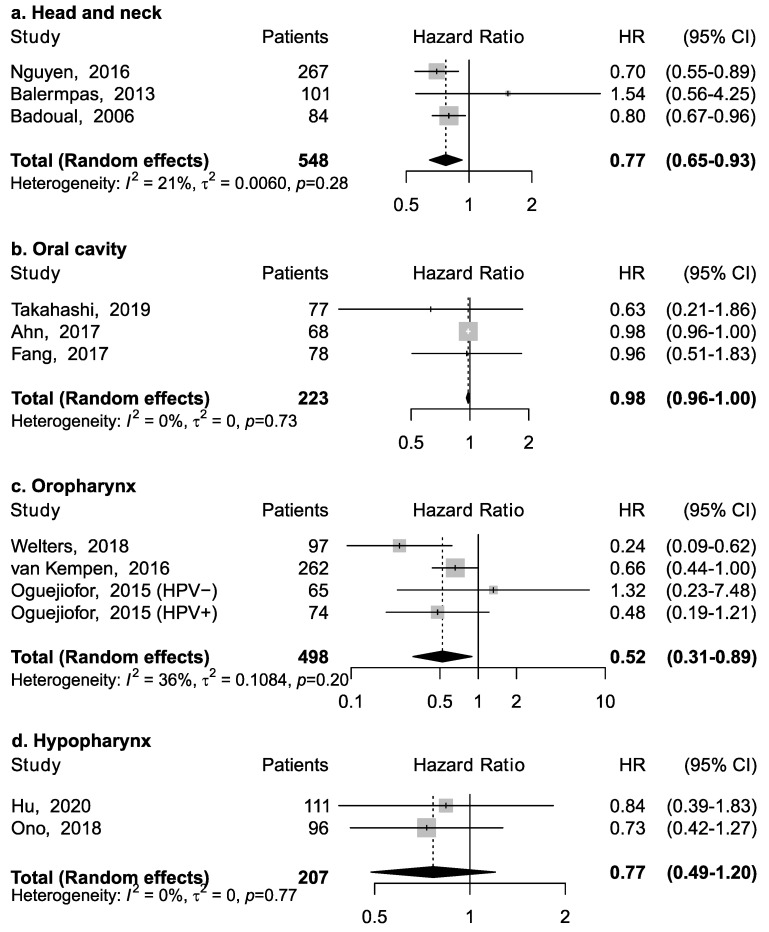
Forest plots for the association between CD4+ tumor-infiltrating lymphocytes and overall survival in head and neck cancers, according to subsite (**a**–**d**). For each study, hazard ratio (HR) and corresponding 95% confidence interval (CI) are reported; HRs are represented through squares, the area is inversely proportional to standard error; 95% CI are represented through horizontal lines. Pooled HRs are represented through diamonds.

Eleven studies reported the association between CD4+ and overall survival (Figure 2). Three studies reported results for head and neck cancers without information on specific anatomical subsite; among them, the pooled HR was 0.77 (95% CI: 0.65–0.93), suggesting an inverse association between CD4+ and mortality (Figure 2a). However, this association appeared to vary according to specific subsite (Figure 2b–d). CD4+ was associated with a lower risk of death for cancer of the oropharynx (pooled HR = 0.52; 95% CI: 0.31–0.89), but not of the oral cavity (pooled HR = 0.98; 95% CI: 0.96–1.00) and hypopharynx (pooled HR = 0.77; 95% CI: 0.49–1.20). No specific data was available for laryngeal cancer.

### 3.2. CD8+ and Overall Survival

**Figure 3:**
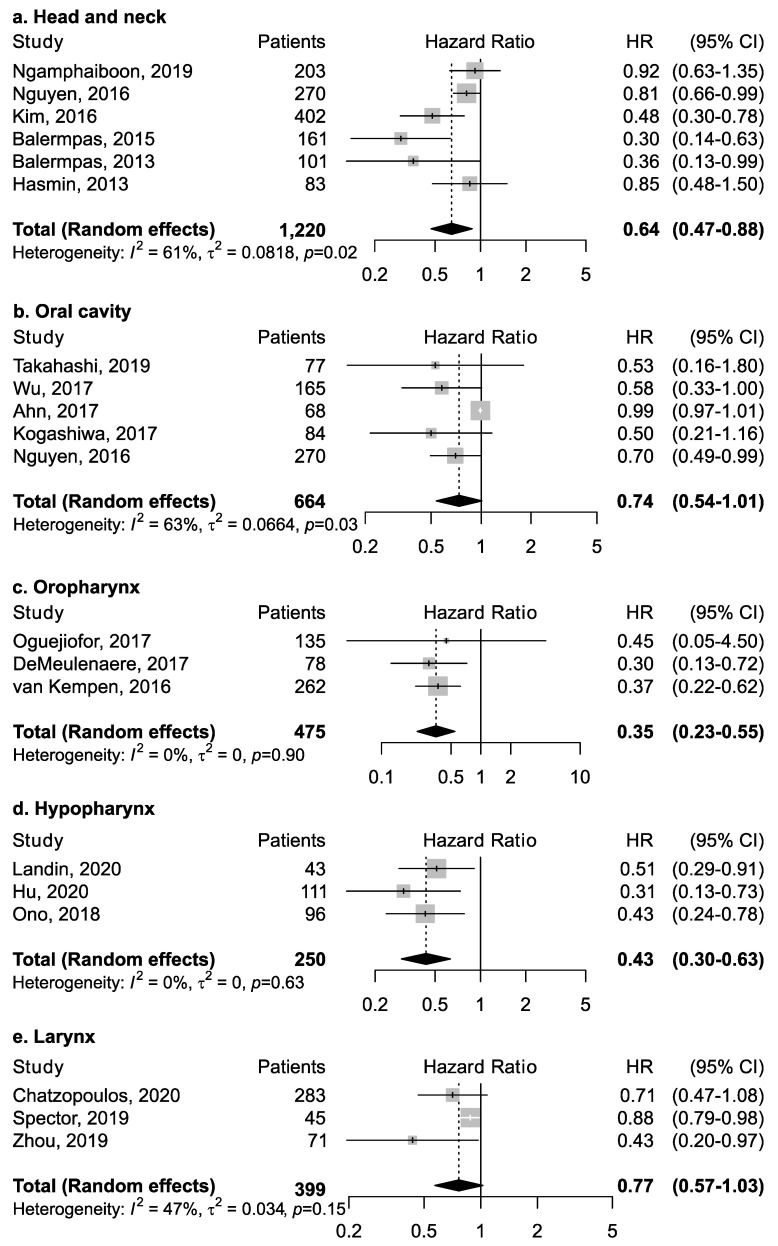
Forest plots for the association between CD8+ tumor-infiltrating lymphocytes and overall survival in head and neck cancers, according to subsite (**a**–**e**). For each study, hazard ratio (HR) and corresponding 95% confidence interval (CI) are reported; HRs are represented through squares, the area is inversely proportional to standard error; 95% CI are represented through horizontal lines. Pooled HRs are represented through diamonds.

Twenty-five studies investigated the association between CD8+ and overall survival (Table 1). Among studies that reported results for all head and neck cancer subsites combined (Figure 3), CD8+ was associated with increased survival (pooled HR = 0.64; 95% CI: 0.47–0.88). No publication bias emerged by funnel plot inspection; further, influence analysis reported pooled HR ranging from 0.57 (CI: 0.37–0.89) by excluding the study by Nguyen et al. [32] to 0.73 (CI: 0.57–0.94) by excluding the study by Balermpas et al. [15].

**Figure 4:**
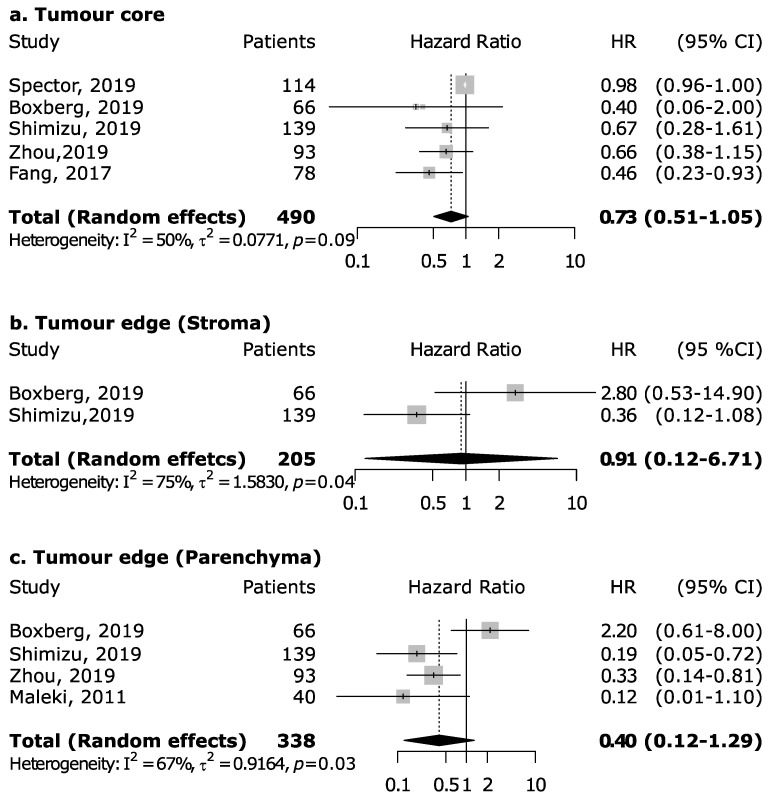
Forest plots for the association between CD8+ tumor-infiltrating lymphocytes and overall survival in oral cancer according to tumor compartment (**a**–**c**). For each study, hazard ratio (HR) and corresponding 95% confidence interval (CI) are reported; HRs are represented through squares, the area is inversely proportional to standard error; 95% CI are represented through horizontal lines. Pooled HRs are represented through diamonds.

**Figure 5:**
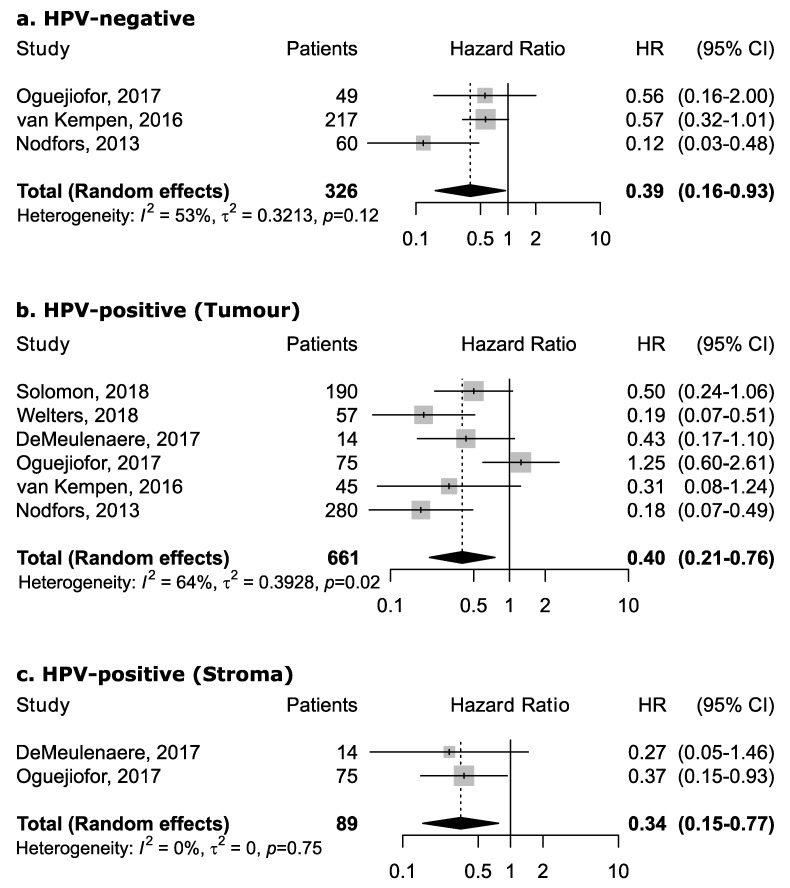
Forest plots for the association between CD8+ tumor-infiltrating lymphocytes and overall survival in oropharyngeal cancer according to HPV status and tumor compartment (**a**–**c**). For each study, hazard ratio (HR) and corresponding 95% confidence interval (CI) are reported; HRs are represented through squares, the area is inversely proportional to standard error; 95% CI are represented through horizontal lines. Pooled HRs are represented through diamonds.

However, the magnitude of this association may depend upon the proportion of the specific intra-tumoral subsite data included in each study, since the prognostic relevance of CD8+ in head and neck cancers was found to vary according to intra-tumoral location. CD8+ in oral cancer trended towards improved survival (pooled HR 0.74, 95% CI: 0.54–1.01) although not to a statistically significant level. Results for intra-tumoral subsite and CD8+ in oral cavity cancers appeared largely heterogenous (Figure 4)—tumor core and parenchymal CD8+ trended towards an improved survival—while stromal CD8+ was inconclusive. Conversely, CD8+ was strongly associated with increased survival in oropharyngeal cancer (Figure 5), in both HPV-negative (pooled HR = 0.39; 95% CI:0.16–0.93) and HPV-positive cancers (pooled HR = 0.40; 95% CI:0.21–0.76 on tumor samples and pooled HR = 0.34; 95% CI:0.15–0.77 on stromal samples) with HPV status being determined by both p16 and DNA in all studies with exception of study by Solomon et al. [37] which defined HPV positivity based on p16 immunostaining alone. No publication bias emerged by funnel plot inspection for HPV-positive tumor samples. Furthermore, consistent results emerged from influence analysis, with pooled HR ranging from 0.32 (0.21–0.49) by excluding Oguejiofor et al. [33] to 0.47 (0.24–0.91) by excluding Nodfors et al. [16]. Three studies reported results for CD8+ and survival in hypopharyngeal cancer (Figure 3d) showing increased survival with pooled HR = 0.43 (0.30–0.63). In addition, 3 studies reported results for laryngeal cancer (Figure 3e), showing homogeneous associations with increased survival, although not to a significant level (pooled HR = 0.77; 95% CI: 0.57–1.03).

## 4. Discussion

### 4.1. Summary of Results

The present meta-analysis focused on the prognostic role of elevated CD4+ and CD8+ TIL populations in HNSCC, reporting on 28 studies. Our principal finding is that while CD4+ and CD8+ TIL populations associate with improved OS in HNSCC, there was considerable heterogeneity in outcomes between different tumor anatomical subsites—previously unreported in the literature. We observed that across datasets of pooled HNSCC anatomical subsites, both CD4+ and CD8+ were associated with improved OS. High CD4+ and CD8+ TILs were significantly associated with improved OS among oropharyngeal cancers and high CD8+ was associated with improved OS in hypopharyngeal cancers. HPV status did not differentiate TIL significance in oropharyngeal cancers. In contrast, neither high CD4+ nor CD8+ TILs were associated with improved OS for oral cavity, laryngeal or hypopharyngeal cancers. Trends observed with intra-tumoral CD8+ TIL heterogeneity were inconclusive due to limited studies and anatomical subsite datasets.

### 4.2. Clinical Significance

Several studies have reported the presence of TILs as a favorable prognostic factor for treatment outcomes in different types of cancer [47]. However, different subsets of lymphocytes have different functions within the TIM [14] and have been found to infer differing prognostic significance. A general observation of our data was that patterns of HR significance were maintained among *both* CD4+ and CD8+ TIL populations for anatomical subsites, indicating that tumor location is a greater discriminating factor than T-cell subset in HNSCC.

We are unable to draw any firm conclusions relating to intra-tumoral CD4+/CD8+ TIL location and prognostic significance (Figure 4 and Figure 5). In part due to low numbers of studies, but also heterogeneity between datasets, study criteria, and number of defined intra-tumoral locations. Reports from other cancers, for example colorectal cancer, have highlighted prognostic significance of TIL subsets and intra-tumoral heterogeneity [48]. Nonetheless, such trends should warrant further investigation and the clinical translation of T-cell TIL markers in HNSCC will be dependent upon standardization of such parameters. In particular, as HNSCC exhibits high levels of intra-tumoral heterogeneity, of both the genomic and immune landscape [49].

The HPV status of oropharyngeal cancers is one of very few predictive markers in HNSCC [50]. The recent separation of HPV +ve and −ve disease into two different disease entities in the 8th Edition of the AJCC staging criteria poses several questions. Notably, how can TILs be incorporated into these different prognostic models. In addition, using p16 immunostaining as a stand-alone test to define an oropharyngeal carcinoma as HPV-driven also raises concerns [51]. p16 immunostaining has indeed shown suboptimal sensitivity and insufficient specificity with 10 to 20% of p16-positive oropharyngeal carcinoma resulting HPV-DNA/RNA negative. Thus, prognostic stratification based on p16 alone has been found inadequate with respect to one based on more precise biomarkers of transforming HPV infections [52]. The underlying driver of carcinogenesis also appears to influence the density and composition of the lymphatic infiltrate—as demonstrated in hormone receptor-negative breast cancers which are observed to have higher TIL density than hormone receptor-positive breast cancers [53,54]. Given the differing underlying etiology and carcinogenic pathways in HPV+ve and −ve cancers and the role of the TIM in invasion and metastasis [55], we had expected to see differing TIL significance in these sub-groups. While only CD8+ datasets were available, the prognostic significance remained within both sub-groups. It remains to be seen what prognostic/predictive value other TIL phenotypic subsets may hold in the HPV+ve tumor sub-group.

### 4.3. Future Work

To incorporate TIL biomarkers, and specifically T-cell subset markers, into clinical practice it is crucial to establish standardized assessment protocols and general cut-off values. As previously highlighted, consensus is forming with regard how to assess TILs as a whole; however, evidence for phenotypic subset quantification is lacking in HNSCC. For the clinical translation of these biomarkers, such standardized protocols are the first step to design robust randomized clinical trials to enable more accurate patient treatment stratification. Results in other cancer types that indicate an equivalent if not improved prognostic value of TILs as biomarkers when compared to conventional TNM classification systems are certainly promising for the incorporation of TILs into existing HNSCC prognostic models [56,57].

To gain a deeper understanding of the TIM, further characterization is needed, including identification of phenotypically distinct immune cell populations and their state of activation or exhaustion. Novel development of multi-parameter assessment methods to view several cell types and corresponding marker expression may facilitate these goals. Such as multiplex fluorescence immunohistochemistry, which is a promising technique that has the ability to simultaneously assess multiple cell subsets in situ, maximize data harvesting per tissue section, improve the quality and detail of pathological analysis and thus efficient tissue use [58]. Finally, at present it is unclear whether conventional treatment protocols for HNSCC inhibit or promote tumor immune response mechanisms. Distel and Buttner [59] investigated intra-tumoral immune profiles before and after primary chemoradiation and concluded that post-therapy cytotoxic T lymphocytes were depleted to a lesser extent than immunosuppressive T regulatory cells. Such findings will influence the timing of TIL assessment in conventional treatment pathways and the clinical efficacy of such values should pre-defined cut-offs be universally endorsed.

The primary recommendation from this meta-analysis is the need for further studies that investigate homogenous patient and/or treatment groups, for example immune-checkpoint inhibitors, with clearly defined research questions. Key areas of interest will be immune cell intra-tumoral heterogeneity and the relationship of therapeutic target marker expression, such as programmed death-ligand 1, to TILs and in particular CD4+ and CD8+ T-cell subsets. Immunotherapy in HNSCC is poorly served by predictive biomarkers, thus the addition of TIL scores to more accurately stratify these patients may hold particular promise.

### 4.4. Limitations

This study has some limitations. Although the number of papers included in this study are adequate, some studies did not provide detailed information regarding the subtypes of TILs or each tumor subsite. In addition, specific treatment groups within studies were heterogenous or poorly reported and thus not amendable to individual analysis. The prognostic value of TIL biomarkers is likely to differ between different tumor subsites, tumor stage, and treatment groups. Due to different cut-off values of the high-density and low-density groups of TILs, techniques of detecting TILs, and the source of specimen, the level of interstudy heterogeneity was relatively high.

## 5. Conclusions

We have demonstrated that high-density CD4+ and CD8+ TILs were associated with improved OS rates in HNSCC. Moreover, tumor location and intra-tumoral subsite significantly influenced the prognostic value of these TIL biomarkers—with a clear separation between oral cavity and oropharyngeal cancers. Our data clearly demonstrates the requirement for well-designed prospective studies with the aim of generating standardized TIL assessment protocols for clinical translation.

## Figures and Tables

**Table 1:** Characteristics and quality assessment of included studies.

Study	Country	Enrolment Period	Sample Size	Subsite	TILs	Newcastle–Ottawa Scale
Chatzopoulos, 2020 [23]	Greece	1985–2008	283	Larynx	CD8	8
Hu 2020 [27]	China	2014–2017	111	Hypopharynx	CD4, CD8	8
Landin 2020 [29]	Sweden	2002–2013	149	Hypopharynx	CD8	8
Boxberg, 2019 [46]	Germany	2008–2012	66	Oral cavity	CD8	6
Ngamphaiboon, 2019 [31]	Thailand	2007–2013	203	Head & neck	CD8	8
Shimizu, 2019 [36]	Japan	2004–2014	139	Oral cavity	CD8	8
Spector, 2019 [38]	U. S. A.	2008–2014	234	Head & neck, oral cavity, larynx	CD8	8
Takahashi, 2019 [39]	Japan	2000–2012	77	Oral cavity	CD4, CD8	5
Zhou, 2019 [43]	China	2004–2015	164	Oral cavity, larynx	CD8	7
Ono, 2018 [35]	Japan	2000–2014	96	Hypopharynx	CD4	8
Solomon, 2018 [37]	Australia	2002–2012	190	Oropharynx HPV^+^	CD8	8
Welters, 2018 [41]	The Netherlands	2007–2015	97	Oropharynx HPV^+^	CD4, CD8	6
Ahn, 2017 [21]	South Korea	2003–2011	68	Oral cavity	CD4, CD8	8
DeMeulenaere, 2017 [24]	Belgium	2004–2013	14	Oropharynx HPV^+^	CD8	8
Fang, 2017 [25]	China	2007–2009	78	Oral cavity	CD4, CD8	8
Kogashiwa, 2017 [44]	Japan	2007–2014	84	Oral cavity	CD8	8
Oguejofor, 2017 [33]	U. K.	2002–2011	124	Oropharynx HPV^−/+^	CD8	8
Wu, 2017 [42]	China	2008–2015	165	Oral cavity	CD8	8
Kim, 2016 [28]	South Korea	2005–2012	402	Head & neck	CD8	8
Nguyen, 2016 [32]	U. S. A.	2008–2012	270	Head & neck, oral cavity	CD4, CD8	7
van Kempen, 2016 [40]	The Netherlands	1997–2011	262	Oropharynx HPV^−/+^	CD4, CD8	8
Balermpas, 2015 [15]	Germany	2004–2012	161	Head & neck	CD8	8
Oguejofor, 2015 [34]	U. K.	2002–2011	139	Oropharynx HPV^−^	CD4	7
Balermpas, 2013 [15]	Germany	2007–2010	101	Head & neck	CD4, CD8	6
Hasmin, 2013 [26]	France	2004–2011	83	Head & neck	CD8	8
Nodfors, 2013 [16]	Sweden	2000–2007	340	Oropharynx HPV^−/+^	CD8	8
Maleki, 2011 [30]	U. S. A.	---	40	Oral cavity	CD8	5
Badoual, 2006 [22]	France	---	84	Head & neck	CD4	7

## Supplementary Materials

The following are available online at https://www.mdpi.com/2072-6694/13/4/781/s1, Table S1: Newcastle-Ottowa Scale assessment of quality of included studies.
