# Supplementary material for: Prognostic Significance of CD4+ and CD8+ Tumor-Infiltrating Lymphocytes in Head and Neck Squamous Cell Carcinoma: A Meta-Analysis
Source: Cancers (Basel). 2021 Feb 13;13(4):781. doi: 10.3390/cancers13040781 (PMC7918220; doi:10.3390/cancers13040781)
Supplement: Supplementary file 1 [file cancers-13-00781-s001.pdf]

Table S1: Newcastle-Ottowa Scale assessment of quality of included studies.

Study	Selection				Comparability	Outcome			Total
	Representativeness of the exposed cohort	Selection of the non-exposed cohort	Ascertainment of exposure	Demonstration that outcome of interest was not present at start of study	Comparability of cohorts on the basis of the design or analysis controlled for confounders	Assessment of outcome	Was follow-up long enough for outcomes to occur	Adequacy of follow up of cohorts	
Chatzopoulos, 2020	*	*	*	*	*	*	*	*	8
Hu 2020	*	*	*	*	*	*	*	*	8
Landin 2020	*	*	*	*	*	*	*	*	8
Boxberg, 2019	-	*	*	*	*	*	-	*	6
Ngamphaiboon, 2019	*	*	*	*	*	*	*	*	8
Shimizu, 2019	*	*	*	*	*	*	*	*	8
Spector, 2019	*	*	*	*	*	*	*	*	8
Takahashi, 2019	-	-	*	*	*	*	-	*	5
Zhou, 2019	*	*	*	*	*	*	--	*	7
Ono, 2018	*	*	*	*	*	*	*	*	8
Solomon, 2018	*	*	*	*	*	*	*	*	8
Welter, 2018	*	*	*	*	*	-	-	*	6
Ahn, 2017	*	*	*	*	*	*	*	*	8
De Meulenaere, 2017	*	*	*	*	*	*	*	*	8
Fang, 2017	*	*	*	*	*	*	*	*	8
Kogashiwa, 2017	*	*	*	*	*	*	*	*	8
Oguejiofor, 2017	*	*	*	*	*	-	*	*	8
Wu, 2017	*	*	*	*	*	*	*	*	8
Kim, 2016	*	*	*	*	*	*	*	*	8
Nguyen, 2016	*		*	*	*	*	*	*	7
van Kempen, 2016	*	*	*	*	*	*	*	*	8
Balermpas, 2015	*	*	*	*	*	*	*	*	8
Oguejiofor, 2015	*	*	*	*	*	-	*	*	7
Balermpas, 2013	*		*	*	*	*	--	*	6
Hasmin, 2013	*	*	*	*	*	*	*	*	8
Nodfors, 2013	*	*	*	*	*	*	*	*	8
Maleki, 2011	-	*	*	*	*	-	-	*	5
Badoual, 2006	*	*	*	*	*	*	--	*	7
